# *Clinical Neuroradiology*: Challenges and Perspectives

**DOI:** 10.1007/s00062-022-01208-4

**Published:** 2022-08-29

**Authors:** László Solymosi

**Affiliations:** Dept. of Neuroradiology, Universitätsklinikum Würzburg, Würzburg, Germany

**Fig. 1:**
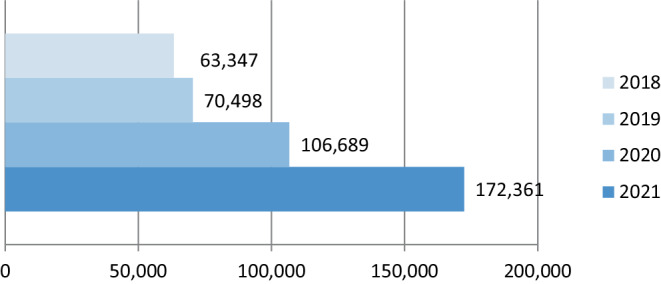
Development of article accesses at *Clinical Neuroradiology* in recent years

**Fig. 2:**
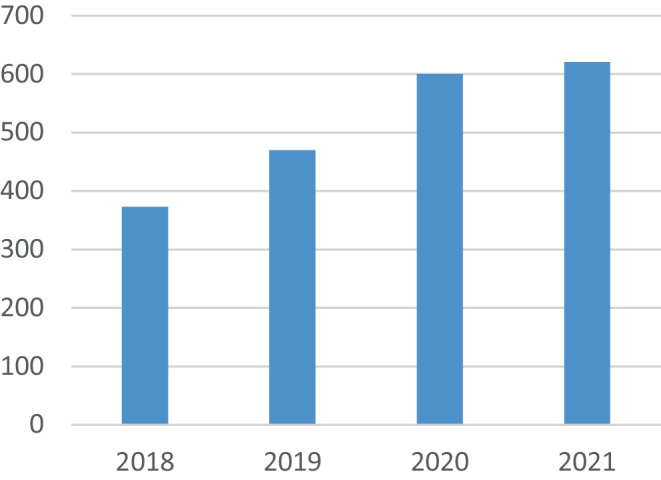
Development of article submissions to *Clinical Neuroradiology* in recent years

Last year, we reported on the enormous success *Clinical Neuroradiology* had seen over the course of the past three decades [1]. The journal has seen a continuous increase in both article accesses (Fig. 1) and article submissions (Fig. 2), showing the increasing interest of the research community in our publications both as authors and readers.

Journal metrics, such as article accesses and submissions, showcase a journal’s development within its field; as such, the journal impact factor receives much attention as one of the most prominent journal metrics.

**Fig. 3:**
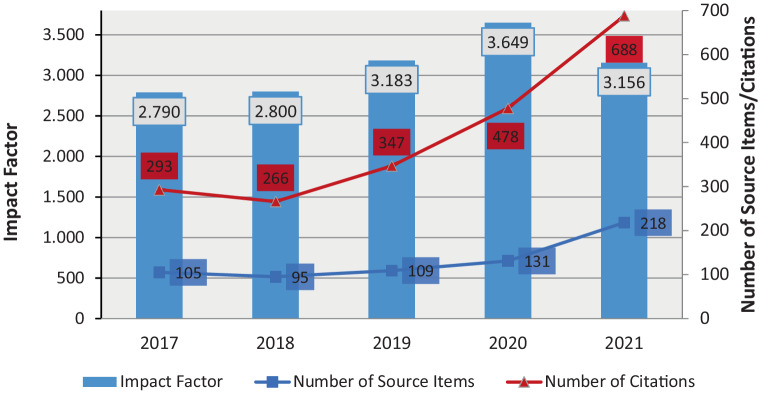
Development of the journal impact factor of *Clinical Neuroradiology* in recent years

While the journal impact factor of *Clinical Neuroradiology* has risen continuously in recent years, we are seeing a drop for the first time on publication of the journal impact factors in mid-2022 (Fig. 3):

While the number of citations relevant for the journal impact factor 2021 increased by 44% in comparison to the previous year, the increase in the number of published items was even greater with around 66%.

This is not only due to a higher number of quality papers accepted by the journal, but also a reflection of the changes in impact factor calculation undertaken in recent years by Clarivate: For the first time, the early access content from the 2020 data was incorporated into the denominator of the Journal Impact Factor (JIF), the number of citable items, while additional citations stem from the inclusion of additional early access content in 2021 contributing to the numerator [2].

This change in calculation, along with the enormous impact of COVID-19 on publications and citations has caused many shifts in citation numbers and rankings. While this has affected a dip in impact factor and ranking for *Clinical Neuroradiology* this year, we are positive that the quality of our submissions and publications continues to increase.

We are looking forward to your continued support of the journal as readers, authors and reviewers and will take all measures to continue to offer a just and profound peer review process and to serve as an excellent publication home for research of the international neuroradiological community.

László Solymosi

Editor-in-Chief
